# Male and female brain evolution is subject to contrasting selection pressures in primates

**DOI:** 10.1186/1741-7007-5-21

**Published:** 2007-05-10

**Authors:** Robin IM Dunbar

**Affiliations:** 1 British Academy Centenary Research Project, School of Biological Sciences, University of Liverpool, Liverpool L69 7ZB, UK

## Abstract

The claim that differences in brain size across primate species has mainly been driven by the demands of sociality (the "social brain" hypothesis) is now widely accepted. Some of the evidence to support this comes from the fact that species that live in large social groups have larger brains, and in particular larger neocortices. Lindenfors and colleagues (*BMC Biology *5:20) add significantly to our appreciation of this process by showing that there are striking differences between the two sexes in the social mechanisms and brain units involved. Female sociality (which is more affiliative) is related most closely to neocortex volume, but male sociality (which is more competitive and combative) is more closely related to subcortical units (notably those associated with emotional responses). Thus different brain units have responded to different selection pressures.

Lindenfors and colleagues [1] provide us with an elegant example of how to use comparative methods to test complex hypotheses so as to reveal something novel and previously unappreciated about the world. They argue that sex differences in the nature of sociality might mean that the brains of the two sexes have been influenced by different selection pressures. In primates, the intensity of social life imposes massive cognitive requirements in a way that is probably unique to this order. Nonetheless, sexual selection is a powerful influence on male behaviour and anatomy [2], and this raises the question of whether it is conflict or cooperation that underpins primate sociality. Testing between these hypotheses directly is all but impossible, as none of the brain databases available provide sufficiently large samples for the two sexes to be separated. Instead, Lindenfors et al make the novel suggestion that we might gain some insights by asking whether simple indices of the costs of sociality (typical number of females in a group) and sexual selection (number of males) correlate differentially with different brain units.

This study provides a nice example of a kind of analysis that could not have been performed other than by using the comparative methods of evolutionary biology. The past decade has witnessed the rise of two quite separate approaches to the study of brain evolution. One derives from evolutionary biology, and uses cross-species analyses to test statistical hypotheses; the other derives from neuroscience, and uses single-species descriptions or experimental tests comparing (usually) just two or three species.

The discovery of novel cell units (notably spindle cells in the Anterior Cingulate Cortex [3] or mirror neurones [4]) that may be unique to humans and apes, or that are implicated in complex social behaviour, has attracted particular attention. Similarly, specific genes such as *GLUD2 *(a retrogene, responsible for clearing the by-products of neurone firing) [5] and *ASPM *and *microcephalin *(both of which are implicated in brain growth) [6,7] have been heralded as the way ahead because they seem to be specifically associated with the rapid evolution of the unusually large brains and more sophisticated cognitive abilities of humans [8,9].

Exciting as these developments are, they have one serious drawback: they fail to address two key issues of fundamental importance. First, they merely provide explanations for *how *the changes in brain size (and hence cognitive ability) were brought about; they tell us nothing at all about *why *such changes occurred. The second is that, no matter how exciting they may be, small changes in minor bits of wetware do not explain the one really spectacular feature of brain evolution – the sheer size of the brains with which some species saddle themselves. Brains are exceptionally expensive in terms of energy consumption, accounting for 8–10 times more energy per unit mass than the average for the body as a whole [10]. For it to be worthwhile for an organism to grow a large brain, there must be a proportional benefit. The consensus, for primates at least, is that the benefit comes from the capacity to create and maintain complex societies that provide individuals with much more effective means for solving the problems of everyday survival and reproduction (conventionally known as the "social brain" hypothesis) [11,12].

In my experience, there is a widespread perception in the neurosciences that evolutionary hypotheses can best be tested by comparing two species at the micro-neurobiology level (better still by a transgenic experiment). Although we should not underestimate the importance of such studies, these are at best rather weak tests of any underlying hypotheses. A comparison of two species that differ in some gross trait (e.g. monogamous prairie voles versus polygamous montane voles [13]) hardly comes close to being a serious attempt to control for the vast arrays of behaviour and physiology by which any two species differ. At worst, it falls foul of the classic logical error of inferring *all *from *some *(the well known "composition fallacy"). Most biological phenomena are quantitative, and only a "dose-response" design is really acceptable.

In practice, there is only one way to test hypotheses of this kind, and that is by comparative analyses using modern statistical methods. These have two advantages that experimental manipulations simply cannot hope to match. First, they can draw on very large samples of species. This is important because it allows us to cope with the inevitably large amount of error variance present in all biological phenomena. Second, statistical methods allow us to incorporate many confounding variables at the same time in such a way as to partial out their effects [12]. Experimental manipulations are intended to spread the noise in natural phenomena evenly across experimental conditions, the underlying assumption being that biological processes are univariate and everything else is irrelevant noise [14]. In fact, most biological phenomena are multivariate, and the "noise" is not noise, but rather part of the organism's response to the complexities of the real world. The experimental method was designed to deal with very simple systems; with complex systems, they often destroy the very thing under study [14].

In the present case, Lindenfors et al demonstrate, with appropriate controls for phylogenetic relatedness (always a problem in comparative analyses), that the number of females in a primate group correlates positively only with the relative size of the telencephalon (controlling for total brain volume), and especially with neocortex volume, and not with the volume of other major brain units. In contrast, the number of males correlates positively only with the relative size of the diencephalon (which includes the limbic system and the hypothalamus). Significantly, the septum (which plays a role in controlling aggression) correlates negatively with male group size, suggesting that, as the level of competition between males increases, the level of cortical control over aggression is actually reduced (presumably in order to ensure that males respond aggressively, rather than submissively, to challenges).

This suggests that male and female brains have responded to different kinds of social pressures: females to social integration, males to male-male competition and fighting. This concurs nicely with the evidence from genomic imprinting that neocortex size (broadly considered to be the brain unit most relevant to sociality) is inherited maternally, but the limbic system (perhaps most closely involved in male responsiveness in agonistic contexts) is paternally inherited [15].

These findings address another dispute that has been rumbling on for more than a decade. Developmental neurobiologists [16,17] have been impressed by the degree of scaling between brain components, and have argued that this is an inevitable consequence of the processes of brain ontogeny. In contrast, evolutionary biologists have argued for a mosaic view of brain evolution: individual components can evolve at different rates under different selection pressures [18]. The present findings clearly support the latter claim.

Nonetheless, the bottom line is, perhaps, that we need much closer integration than hitherto seems to have been the case between those who work on the neurobiology and those who work on the larger comparative scale, and a better understanding by both sides of each other's contributions. The current stand-off between neuroscientists and comparative biologists is unproductive for both sides. This is especially true in the context of primate brain evolution, because there has been a consistent (if perhaps understandable) tendency for neuroscientists to use naively simplistic measures of sociality.
